# Influence of lecithin cholesterol acyltransferase alteration during different pathophysiologic conditions: A 45 years bibliometrics analysis

**DOI:** 10.3389/fphar.2022.1062249

**Published:** 2022-12-14

**Authors:** Hongliang Gao, Jing Wu, Zhenyu Sun, Furong Zhang, Tianshu Shi, Ke Lu, Dongfu Qian, Zicheng Yin, Yinjuan Zhao, Jian Qin, Bin Xue

**Affiliations:** ^1^ Core Laboratory, Sir Run Run Hospital, Nanjing Medical University, Nanjing, China; ^2^ School of Clinical Medicine, Wannan Medical College, Wuhu, China; ^3^ Collaborative Innovation Center of Sustainable Forestry in Southern China, College of Forestry, Nanjing Forestry University, Nanjing, China; ^4^ School of Health Policy and Management, Center for Global Health, Nanjing Medical University, Nanjing, China; ^5^ State Key Laboratory of Pharmaceutical Biotechnology, Department of Sports Medicine and Adult Reconstructive Surgery, Nanjing Drum Tower Hospital, The Affiliated Hospital of Nanjing University Medical School, Nanjing, China; ^6^ Research Center for Computer-Aided Drug Discovery, Chinese Academy of Sciences, Shenzhen, China; ^7^ Nanjing Foreign Language School, Nanjing, China

**Keywords:** lecithin cholesterol acyltransferase, physiological, emerging trends, risk, bibliometrics

## Abstract

**Background:** Lecithin cholesterol acyltransferase (LCAT) is an important enzyme responsible for free cholesterol (FC) esterification, which is critical for high density lipoprotein (HDL) maturation and the completion of the reverse cholesterol transport (RCT) process. Plasma LCAT activity and concentration showed various patterns under different physiological and pathological conditions. Research on LCAT has grown rapidly over the past 50 years, but there are no bibliometric studies summarizing this field as a whole. This study aimed to use the bibliometric analysis to demonstrate the trends in LCAT publications, thus offering a brief perspective with regard to future developments in this field.

**Methods:** We used the Web of Science Core Collection to retrieve LCAT-related studies published from 1975 to 2020. The data were further analyzed in the number of studies, the journal which published the most LCAT-related studies, co-authorship network, co-country network, co-institute network, co-reference and the keywords burst by CiteSpace V 5.7.

**Results:** 2584 publications contained 55,311 references were used to analyzed. The number of included articles fluctuated in each year. We found that Journal of lipid research published the most LCAT-related studies. Among all the authors who work on LCAT, they tend to collaborate with a relatively stable group of collaborators to generate several major authors clusters which Albers, J. published the most studies (*n* = 53). The United States of America contributed the greatest proportion (*n* = 1036) of LCAT-related studies. The LCAT-related studies have been focused on the vascular disease, lecithin-cholesterol acyltransferase reaction, phospholipid, cholesterol efflux, chronic kidney disease, milk fever, nephrotic syndrome, platelet-activating factor acetylhydrolase, reconstituted lpa-i, reverse cholesterol transport. Four main research frontiers in terms of burst strength for LCAT-related studies including “transgenic mice”, “oxidative stress”, “risk”, and “cholesterol metabolism “need more attention.

**Conclusion:** This is the first study that demonstrated the trends and future development in LCAT publications. Further studies should focus on the accurate metabolic process of LCAT dependent or independent of RCT using metabolic marker tracking techniques. It was also well worth to further studying the possibility that LCAT may qualify as a biomarker for risk prediction and clinical treatment.

## Introduction

LCAT is an important enzyme involved in the maintenance of tissue and plasma cholesterol equilibrium *via* RCT (Frohlich et al., 1982). It binds to HDL and is activated to execute transesterification activity by apolipoprotein A-I (apoA-I) (Jones et al., 2009). In humans, the LCAT gene is located on the long arm of chromosome 16 (16q22) with a full length of approximately 4200 base pairs which contain six exons and five introns (Gjone, 1988). The mRNA for LCAT was found almost exclusively in the liver, with minor levels identified in the brain and testes (Saeedi et al., 2015). The mature plasma LCAT is a protein with 416 residues and a molecular mass of approximately 67 kDa, which is more than 20% higher than the predicted protein mass. Piper et al. (2015) reported a 2.65Å crystal structure of the human LCAT and revealed that LCAT belongs to the α/β-hydrolase fold family which has a central domain consisting of a mixed seven-stranded β-sheets with four α-helices, and loops linking the β-strands. Besides this, John Tesmer (Glukhova et al., 2015) also described a low-resolution structure of LCAT which suggests the molecular basis underlying human disease for most of the known LCAT missense mutations.

Glomset developed LCAT in 1962 after noticing a drop in lecithin and FC levels in human plasma. He theorized that plasma has a fatty acid transferase that can transfer ester from lecithin to FC (Glomset, 1962) and suggested that the acyltransferase reaction mediated by LCAT is the major source of the esterified cholesterol of the HDL (Glomset, 1970). In fact, LCAT is responsible for the synthesis of about 70% cholesteryl esters in human plasma (Yamamuro et al., 2020). LCAT has gotten a lot of interest when it comes to controlling physiological lipid metabolism (Tosheska Trajkovska and Topuzovska, 2017). Mutations in both alleles of LCAT gene lead to two syndromes namely, familial LCAT deficiency (FLD), and fish-eye disease (FED) which caused serious clinical symptoms included a decrease in HDL cholesterol level, apo A-I and apo A-II, a decrease in LDL cholesterol level and an increase in FC and apo E (Lucca et al., 2016).

LCAT showed a cardiovascular disease preventive effect during physiological states (Rousset et al., 2009), however, multiple pathological and physio-pathological alterations lead to the change of serum LCAT and the protection diminished subsequently. With the development of high-throughput measurement technologies and the application of metabolomics and proteomics, the relationship between LCAT and more diseases will be revealed. Significant progress in the molecular and functional characterization of LCAT has been made over the last few decades. Given this, it is important to understand the knowledge domain and predict the emerging trends as well of LCAT.

Bibliometrics is a quantitative analysis method based on the external characteristics of documents, which uses mathematical and statistical methods to describe, evaluate and predict the research status, emerging trends, and new developments in science & technology (Cooper, 2015). CiteSpace provides cooperation network analysis (including author, institution, and country), co-keywords network analysis and co-citation network analysis (including reference, author, and journal) (Synnestvedt et al., 2005). The disciplinary intellectual base could be identified according to the clustering results of the co-citation network and further predict the emerging trends and new developments of a subject by observing the citing literature groups and burst keywords. However, to the best of our knowledge, there are no papers reporting the bibliometrics analysis of LCAT-related studies.

In this study, we performed a bibliometrics analysis of all the LCAT-related studies from 1975 to 2020 and help to identify current research trends and hotspots in this filed.

## Materials and methods

### Data collection

Articles were collected from the Web of Science Core Collection (WOSCC), which generates standardized and high-quality academic publication information, and is used extensively for the bibliometric examination of the evolution of scientific issues. Then, these data were converted to txt format and imported into CiteSpace V 5.7 for further analysis.

### Search strategy

We used the WOSCC to retrieve LCAT-related studies published from 1975 to 2020. The topic of the data retrieval strategy was “Lecithin: cholesterol acyltransferase”. The search records were downloaded on 17th June 2022. Each downloaded study included the authors, title, abstract, and descriptors and identifiers and was exported to CiteSpace for further analysis.

### Inclusion criteria

Inclusion criteria were: 1) articles and reviews on LCAT; 2) articles published from 1975 to 2020; and 3) articles retrieved from the Web of Science Core Collection.

### Exclusion criteria

Exclusion criteria were: 1) meeting abstract or proceedings papers or editorial material or book chapter or letter correction; 2) articles not officially published; 3) repeated publications; 4) structure of the thesis is incomplete.

### Visualized analysis

The analysis procedure of CiteSpace is identifying a knowledge domain, collecting data, extracting research front terms, time slicing, selecting threshold, pruning, and merging, lay-outing, visualizing inspection, and verifying pivotal points. Modularity(Q) and Silhouette(S) were used to evaluate the map drawing effect. Q is normalized to the unit interval of (0,1), and Q > 0.3 means that the divided structure is significant; S > 0.5 means that the clustering results are reasonable, S > 0.7, means that the clustering results are efficient and convincing. Half-life describes the rate of aging in the documents, the greater the half-life value, the greater the effective value of the documents. Centrality > 0.1 means that the node plays a key role in the network. Tree Ring History (TRH) represents the citation history of an article, the size of TRH reflects the number of citations, and the thickness of TRH is directly proportional to the frequency of citations in that year. Different colors represent documents of different years, cold color represents past historical documents, warm color represents the latest current documents, and we can interpret the development context and historical evolution process of the scientific domain by observing the gradual change process of the atlas from cold to warm. In addition, dual-map overlays were introduced to show the global visualization of literature growth at a disciplinary level. In dual-map overlays, on the left is the journal distribution where the citing literature is located, which can be regarded as field application; the right side corresponds to the distribution of the cited literature, which can be regarded as a research basis.

## Results

### Publication outputs

**FIGURE 1:**
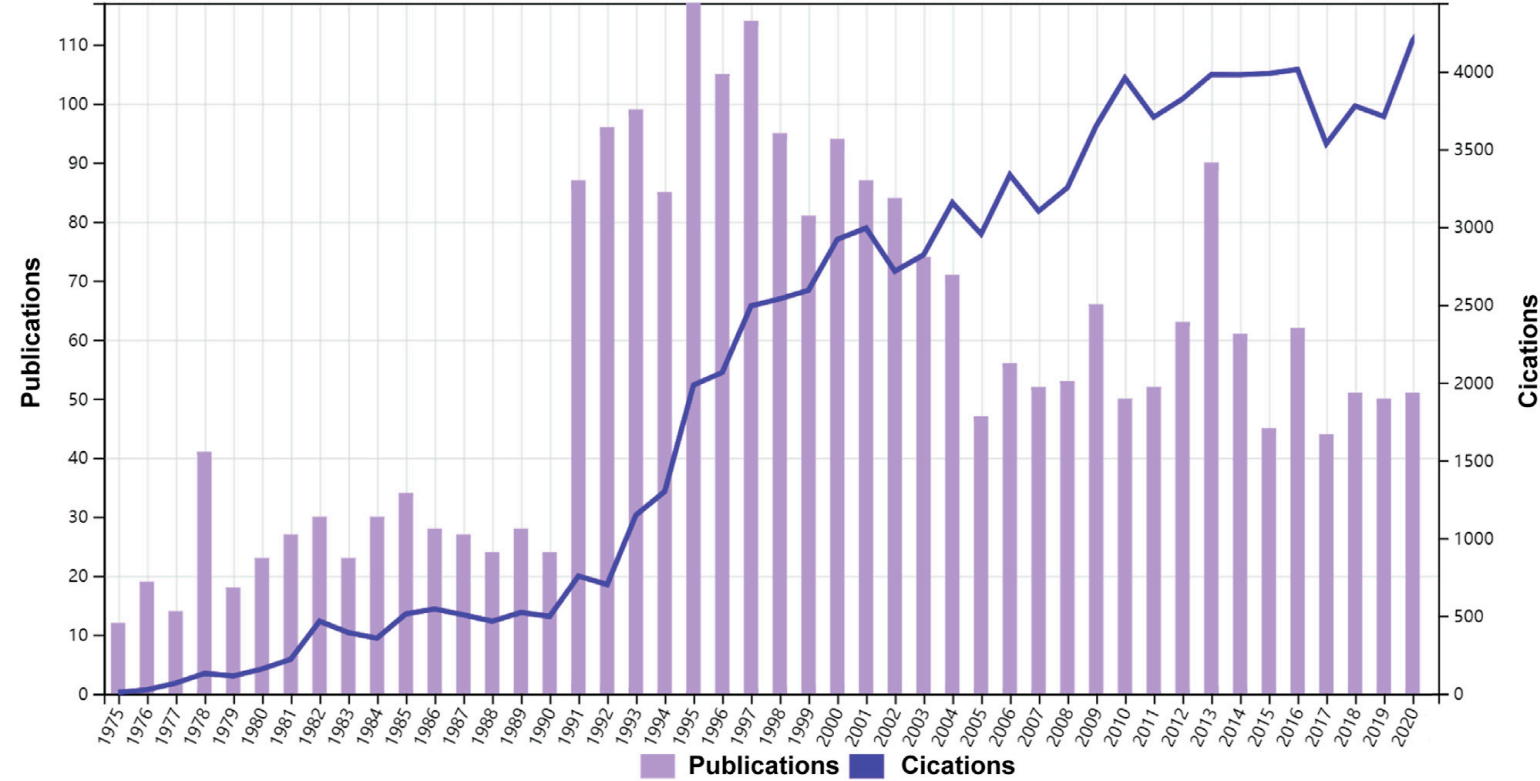
Time sequence of relevant studies on LCAT published from 1975 to 2020 in Web of Science Core Collection.

As shown in Figure 1, 2584 publications contained 2324 articles and 260 reviews met the search strategy. And studies published between 1968 and 1975 was shown in Supplementary Table S1. The total citations were 100,919, and the average citations were 39.04 per item. The first publication on LCAT in the Web of Science database was published on Journal of lipid research in 1966. From that point on, the number of studies on LCAT began to increase rapidly by 2020, a total of 3119 LCAT manuscripts were published. According to the analysis of WOSCC, the number of published studies fluctuated over the studied period and the largest number of articles were published in 1995 with 117 (Figure 1).

### Journal analysis

**FIGURE 2:**
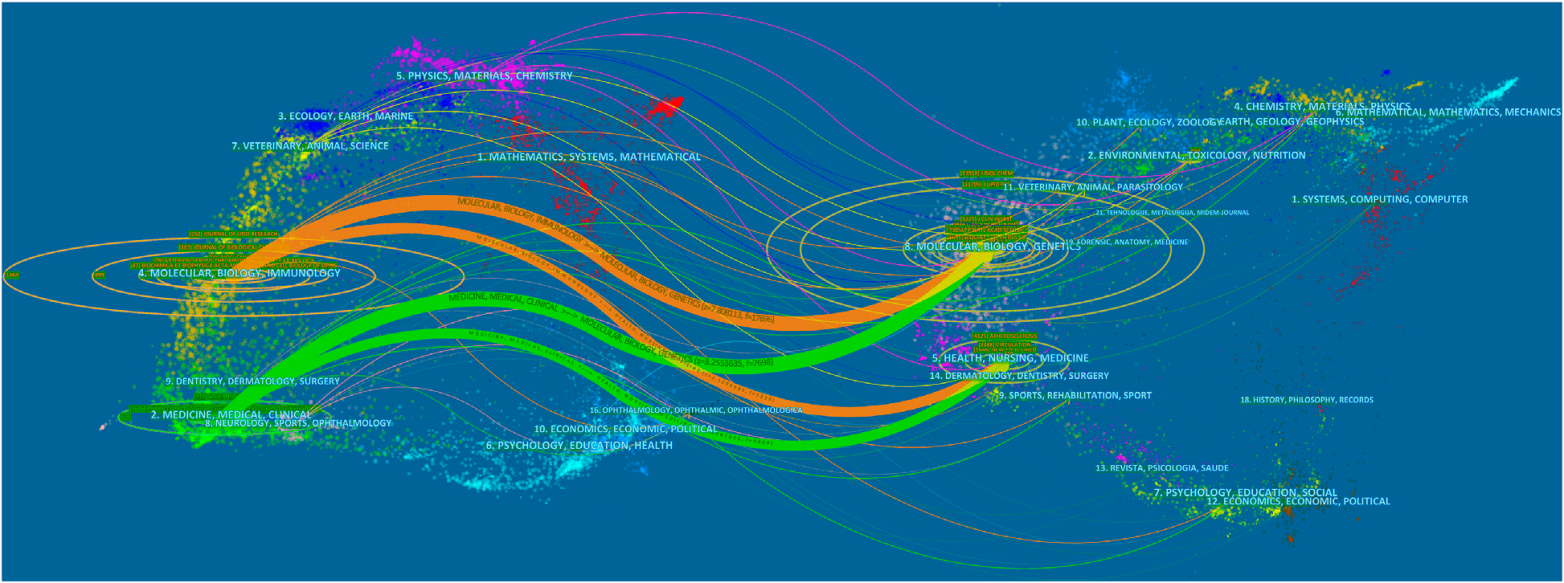
The dual-map overlay of journals related to LCAT studies. The citing journals were on the left, the cited journals were on the right, and the colored path represents the citation relationship.

Studies on LCAT are distributed over hundreds of journals. The ten journals with the largest number of published LCAT studies are listed in Table 1; 251 studies were published by Journal of lipid research. The JLR mainly focused on the science of lipids in health and disease and aims to be at the forefront of genomics, proteomics, and lipidomic as they relate to lipid metabolism and function. It is worth mentioning that LCAT has long been believed to exert an important role in RCT and determine the maturation of HDL. Thus, LCAT has been shown to be closely related to Atherosclerosis. The dual-map overlay of journals stands for the topic distribution of academic journals (Figure 2). Three citation paths colored orange and green were identified, which means the studies published in Molecular/Biology/Genetics journals were mainly cited by the studies published in Molecular/Biology/Immunology journals and Medicine/Medical/Clinical journals. The studies published in Health/Nursing/Medicine journals were mainly cited by Medicine/Medical/Clinical. These can provide important references for new researchers.

**TABLE 1:** Top 10 most productive journals.

Journals		Impact factors	Number of publications published papers
1	*Journal of lipid research*	6.676	251
2	*Journal of biological chemistry*	5.486	163
3	*Atherosclerosis*	6.847	112
4	*Biochemistry*	3.321	98
5	*Arteriosclerosis thrombosis and vascular biology*	10.514	78
6	*Biochimica et biophysica acta*	2.59	76
7	*Biochimica et biophysica acta-molecular and cell biology of lipids*	5.228	47
8	*Clinica chimica acta*	6.314	44
9	*Scandinavian journalof clinical laboratory investigation*	2.209	36
10	*Biochimica et biophysica acta lipids and lipid metabolism*	2.973	35

### Co-country analysis

**FIGURE 3:**
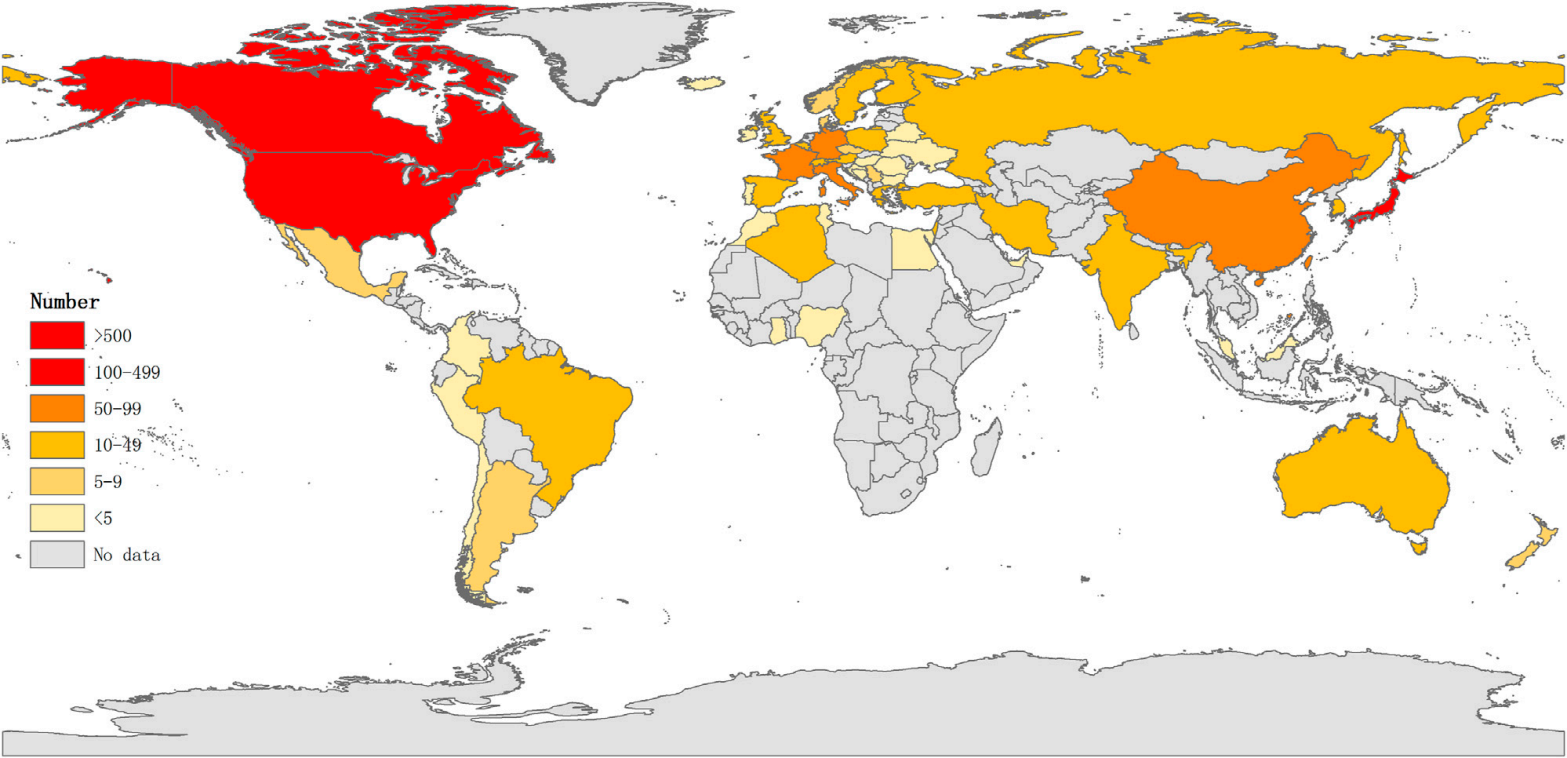
Number of published LCAT-related studies worldwid. The darker the color is, the more the number of published studies.

**FIGURE 4:**
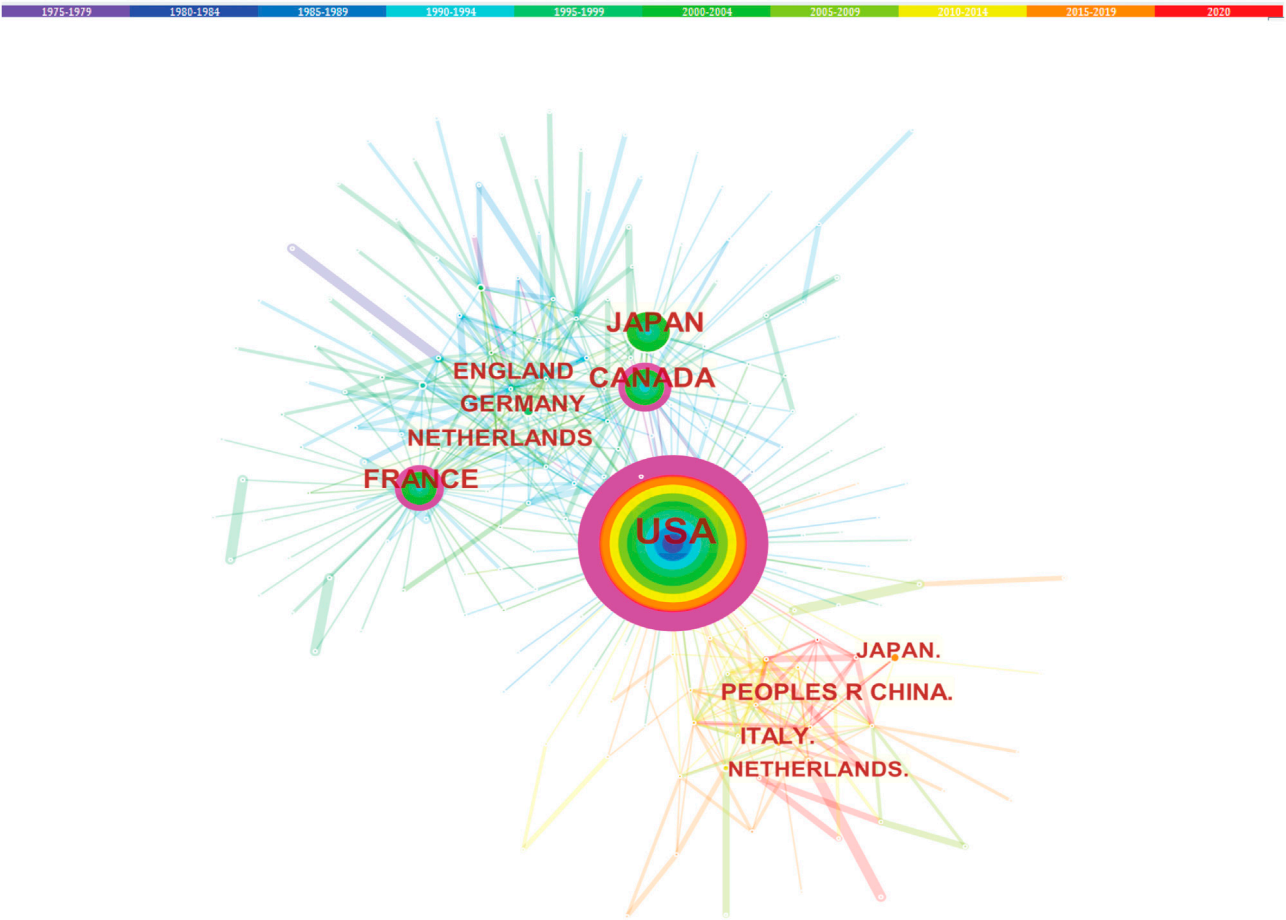
Cooperation among countries. The co-country network among the countries that published the LCAT-related studies.

Studies published from 1975 to 2020 were chosen with a time slice of 5 years for the analysis. The size of the circle represents the number of papers published by the country. The thickness of the purple ring indicates the degree of a country’s centrality, which is a measure associated with the scientific contribution. Number of published LCAT-related studies worldwide is shown in (Figure 3). We found that the United States maintained the dominant position in terms of publications suggesting that the United States exerts a crucial role in this field and cooperation is also existed among countries (Figure 4). United States published 40.1% of LCAT-related studies followed by Japan and Canada (Table 2). The thickness of the line represents cooperation intensity between the two countries. Moreover, a strong collaboration can stimulate the production of high-quality studies.

**TABLE 2:** The Top 10 countries that published studies on LCAT.

	Country	Centrality	Frequency	Percentage (%)	H-INDEX	Total citations
1	United States	1.10	1036	40.1	114	56,520
2	Japan	0.06	292	11.3	45	7,391
3	Canada	0.24	252	9.6	50	8,840
4	France	0.28	189	7.3	47	6,921
5	Germany	0.04	136	5.3	44	6,067
6	Italy	0.05	134	5.2	36	3,771
7	Netherlands	0.09	133	5.1	40	6,157
8	England	0.10	98	3.8	35	3,718
9	China	0.01	85	3.3	24	1,704
10	Australia	0.07	76	3.0	33	3,113

### Co-institute analysis

**FIGURE 5:**
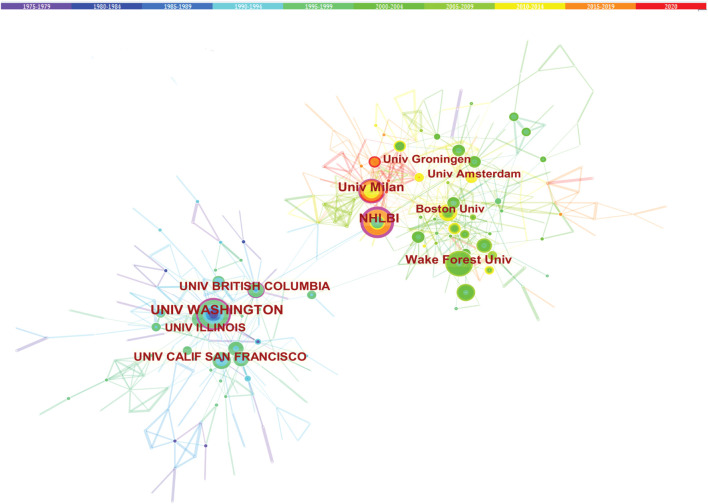
Cooperation among institutions. The co-institute network among the institutions that published the LCAT-related studies.

The cooperation between the two institutions was based on the co-occurrence frequency matrix. Nodes represent institutes, and the size of the circle represents the number of papers published by the institute. These institutions shown in Table 3 are mainly concentrated in higher education research institutions which University of California System published the most studies with 183. The top 10 institutions contributed to 899 articles, accounting for 34.8% of all publications. This is important, as the higher educational institution has played a critical role in LCAT-related studies. Obviously, these institutions tend to cooperate closely and steadily with each other to improve the quality of research results (Figure 5). The institutional distribution provides invaluable information for researchers to identify and choose appropriate collaborative institutions.

**TABLE 3:** The Top 10 institutions that published studies on LCAT.

	Institutes	Frequency (*n* = 2,584)	Percentage (%)	Country
1	University of California System	183	7.1	United States
2	Institut national de la sante et de la researche medical inserm	101	3.9	France
3	University of Washington	88	3.4	United States
4	University of California San francisco	81	3.1	United States
5	University of Illinois System	80	3.1	United States
6	University of British Columbia	78	3.0	England
7	National Institutes of Health	73	2.8	United States
8	Wake forest University	73	2.8	United States
9	Udice French research universities	71	2.7	France
10	University of Milan	71	2.7	Italy

### Co-authorship

**FIGURE 6:**
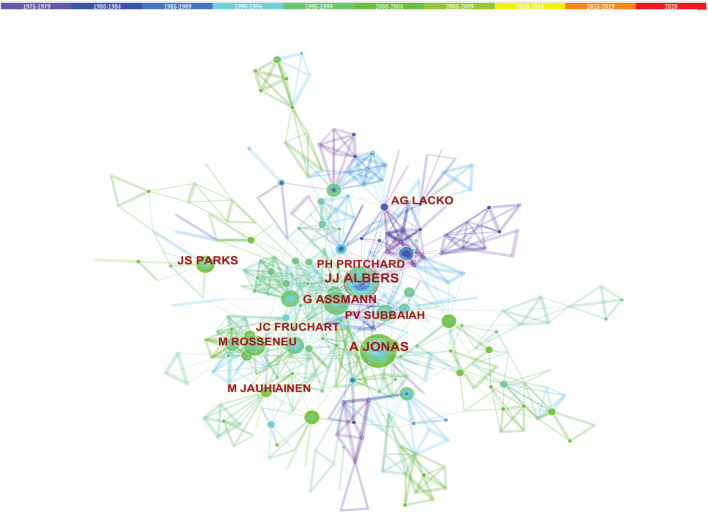
Co-authorship of LCAT-related studies. The co-authorship network that works on LCAT.

Studies published from 1975 to 2020 were chosen with a time slice of 5 years for the analysis, and the selection criteria were the top 10% of most cited or occurred items from per slice. The co-authorship network is displayed in Figure 6. The size of the circle represents the number of studies published by the author. The thickness of the line represents the cooperation intensity between the two authors. The color represents the year of publications, the bluer of the nodes, and the earlier of the publications. Among all the authors who work on LCAT, they tend to collaborate with a relatively stable group of collaborators to generate several major author clusters. Albers, J. published the most articles followed by Jonas, A and van Tol, Arie with 50 and 48 articles respectively (Figure 6).

### Cited reference Co-citation analysis

**FIGURE 7:**
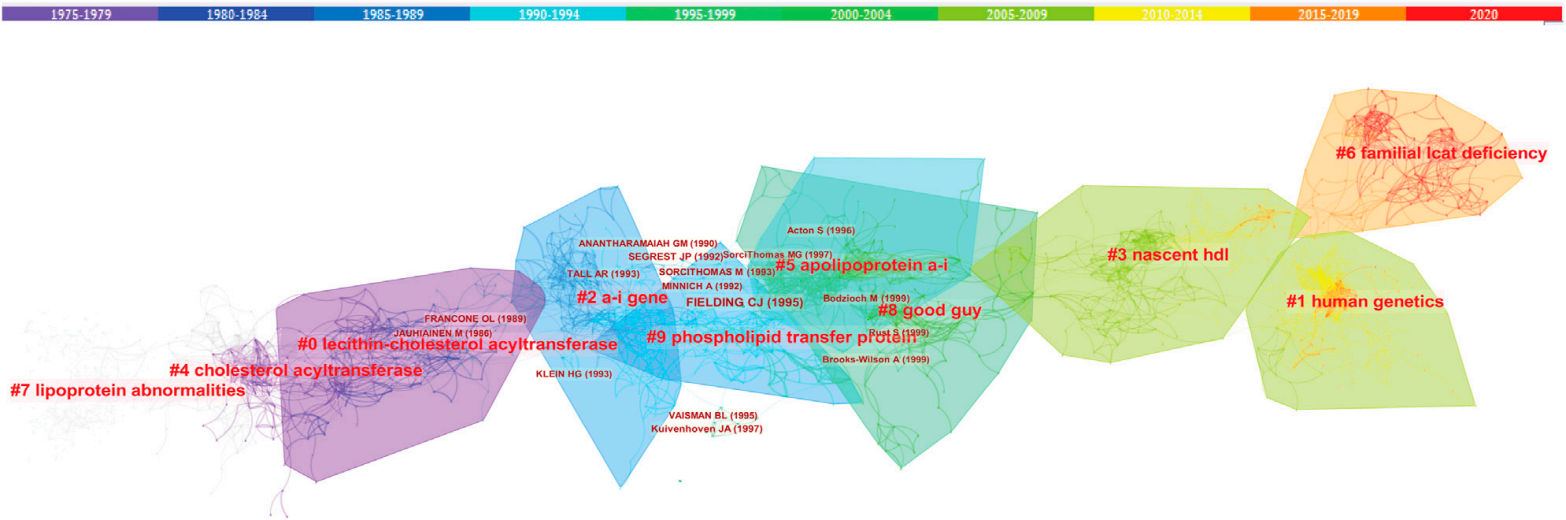
The network of co-cited references clusters. Clusters are labeled in bright red. Key authors are labeled in dark red. The clusters labels and key authors’ labels are located in different outlines with different colors, and the warmer the colors, the closer they are to the latest current. Clusters within the red outline indicate the current research hotspots, that is, rapid increases in citation counts. #, a cluster; hdl, high density lipoprotein.

Trajectories of LCAT-related studies shown in a hybrid network of co-cited references and burst terms are shown in Figure 7. And Table 4 show the top five co-cited references with high centrality.

**TABLE 4:** The top five co-cited references with high centrality.

Rank	First author	Country	Centrality	Year	Cited references	Journal	IF
1	Rien van Haperen	Netherlands	0.19	2000	Human Plasma Phospholipid Transfer Protein Increases the Antiatherogenic Potential of High-Density Lipoproteins in Transgenic Mice	ARTERIOSCL THROM VAS	10.514
2	Angeliki Chroni	UNITED STATES	0.19	2003	The Central Helices of ApoA-I Can Promote ATP-binding Cassette Transporter A1 (ABCA1)-mediated Lipid Efflux	J BIOL CHEM	5.486
3	Dan C. McManus	Canada	0.18	2000	Distinct Central Amphipathic a-Helices in Apolipoprotein A-I Contribute to the *in Vivo* Maturation of High Density Lipoprotein by Either Activating Lecithin-Cholesterol Acyltransferase or Binding Lipids	J BIOL CHEM	5.486
4	Graciela R. Castro	UNITED STATES	0.17	1988	Early incorporation of cell-derived cholesterol into pre-.beta.-migrating high-density lipoprotein	BIOCHEMISTRY-US	3.321
5	Steven E. Nissen	UNITED STATES	0.17	2003	Effect of Recombinant ApoA-I Milano on Coronary Atherosclerosis in Patients With Acute Coronary Syndromes	JAMA-J AM MED ASSOC	157.335

### Co-authorship and cited reference co-citation analysis in the last 10 years

**FIGURE 8:**
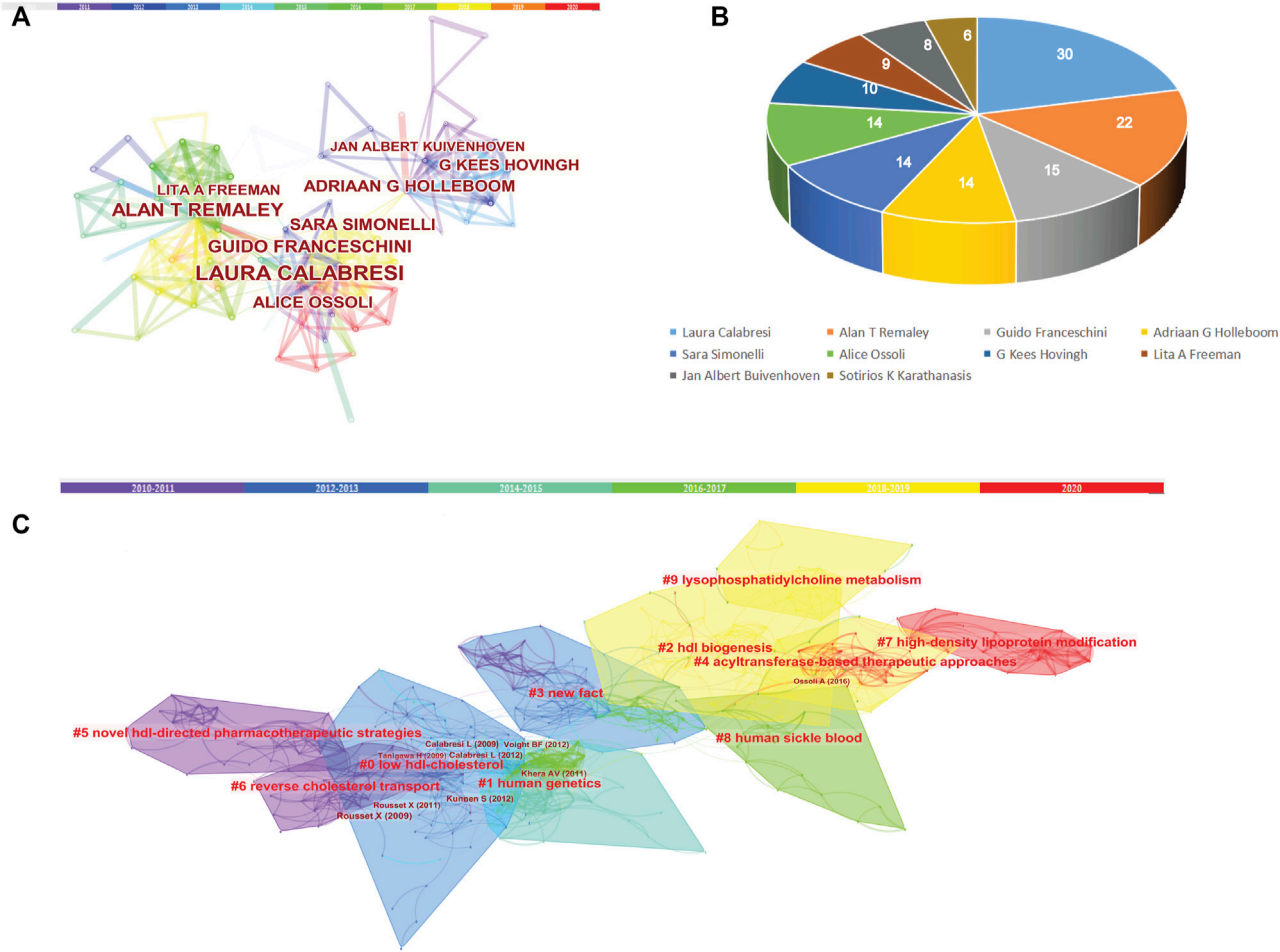
Co-authorship and Cited Reference Co-citation analysis in the last 10 years. **(A)** Co-authorship of LCAT-related studies. **(B)** Authors with the top 10 number of LCAT-related studies. **(C)** The network of co-cited references clusters in the last 10 years.

In order to analyze the author collaboration and contributions in the last 10 years, studies published from 2010 to 2020 were chosen with a time slice of 1 year for the analysis. We found that Laura Calabresi published the most article with 30 and the top 10 most productive authors were shown in Figure 8. In parallel, co-citation analysis showed that the article published by Rouseet X, Calabresi L and Ossoli A et al. have greater influence in LCAT-related studies in the last 10 years (Table 5).

**TABLE 5:** The top five co-cited references with high centrality in the last 10 years.

Rank	First Author	Country	Centrality	Year	Cited references	Journal	IF
1	Amit V Khera	UNITED STATES	0.20	2011	Cholesterol efflux capacity, high-density lipoprotein function, and atherosclerosis	NEW ENGL J MED	91.253
2	Kerry-Anne Rye	Australia	0.15	2014	Regulation of high-density lipoprotein metabolism	CIRC RES	17.367
3	Bronwyn A Kingwell	Australia	0.12	2014	HDL-targeted therapies: progress, failures and future	NAT REV DRUG DISCOV	84.694
4	Rong Huang	UNITED STATES	0.11	2011	Apolipoprotein A-I structural organization in high-density lipoproteins isolated from human plasma	NAT STRUCT MOL BIOL	15.369
5	Laura Calabresi	Italy	0.09	2012	Genetic lecithin:cholesterol acyltransferase deficiency and cardiovascular disease	ATHEROSCLEROSIS	5.162

### Keywords with citation bursts

**FIGURE 9:**
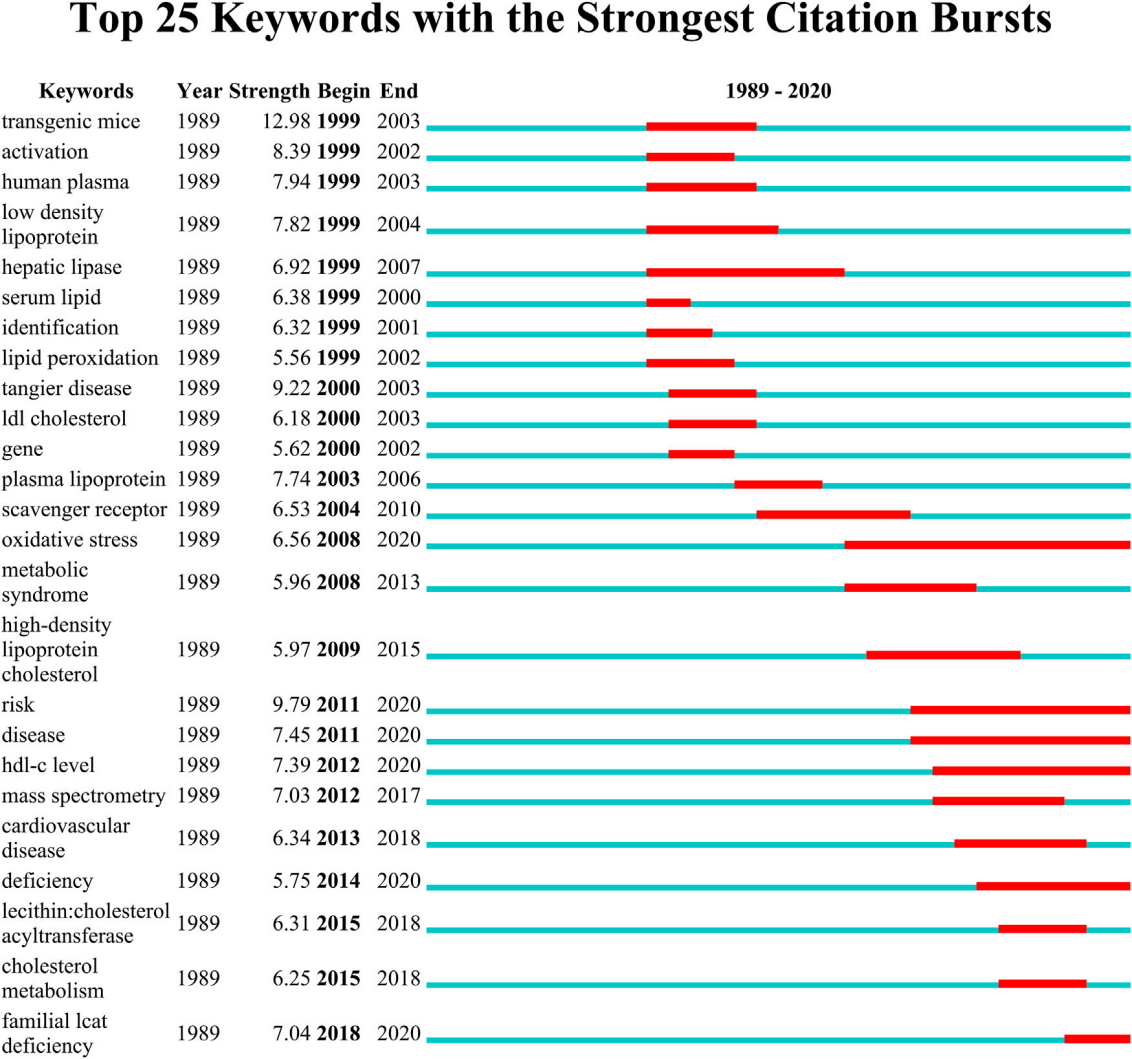
The keywords with the strongest citation bursts for publications on LCAT-related studies from 1985 to 2020. The red line indicates the time period when the keyword had a burst.

Figure 9 shows the top 25 keywords with the strongest citation bursts. The red line indicates the time period when the keyword had a burst. “Transgenic mice” with the strongest citation bursts appeared in 1999, it is thus evident that conducting the study with different transgenic mice such as ApoE^−/−^ and Lcat^−/−^ mice will strengthen the study findings of LCAT. The keywords “oxidative stress”, “risk”, “disease”, “hdl-c level”, “deficiency” and “familial lcat deficiency” with citation bursts extend until 2020.

## Discussion

### Principal results

This study updates current intellectual base on research interests related to LCAT, thus, providing researchers s an overview of LCAT-related studies and potential future research hot spots. We conducted acomprehensive search of literature published on this topic before December 2020 in the Web of Science Core Collection. 55,211 bibliographies were performed a bibliometric analysis by CiteSpace.

### Intellectual base

**FIGURE 10:**
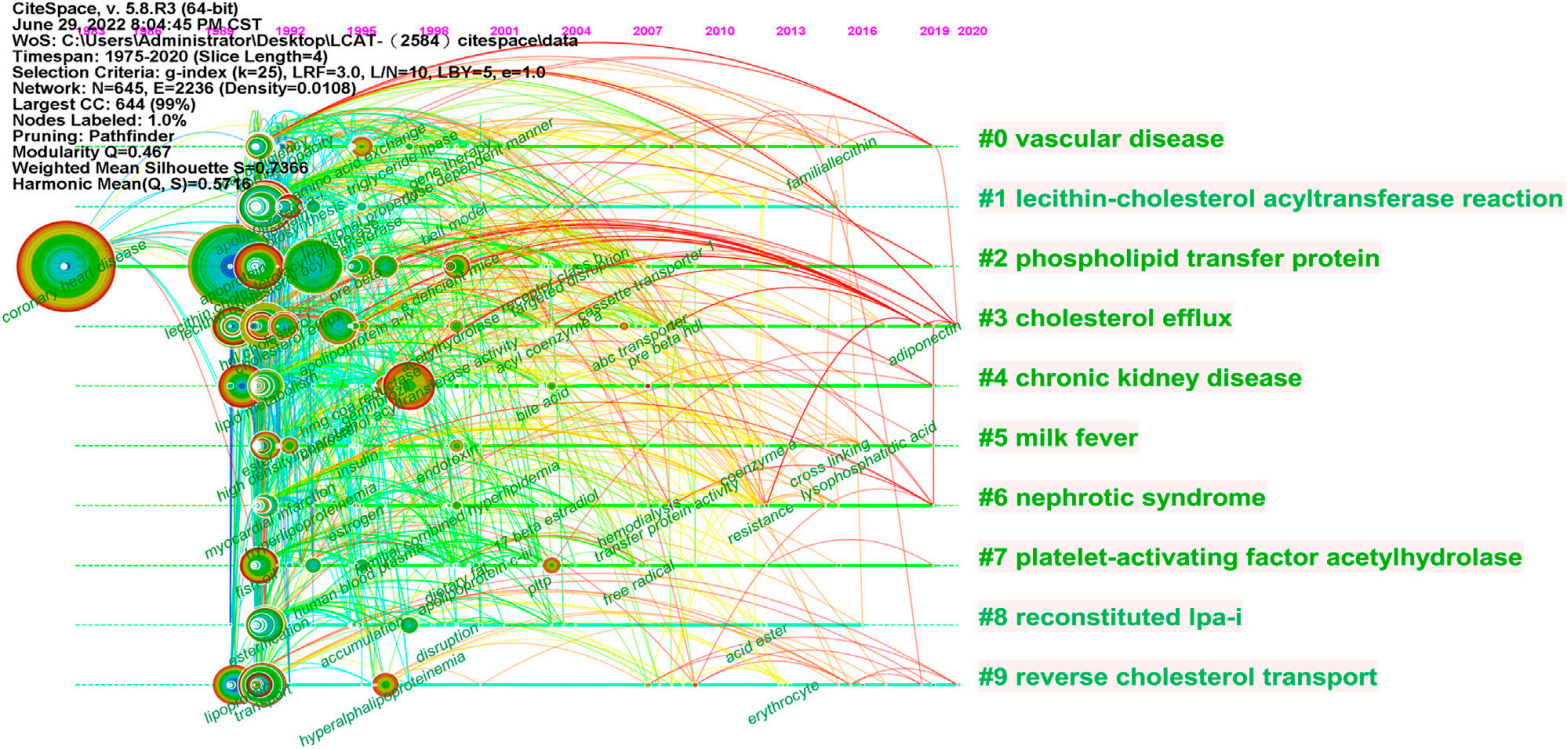
The timeline map of LCAT-related co-citation clusters. Major clusters are labeled on the right. Landmark keywords are labeled on the timelines.

As shown in Figure 10, the LCAT-related studies have been focused on the vascular disease, Lecithin cholesterol acyltransferase reaction, phospholipid transfer protein, cholesterol efflux, chronic kidney disease, milk fever, nephrotic syndrome, platelet-activating factor acetylhydrolase, reconstituded lpa-i, reverse cholesterol transport presented by CiteSpace. Since the important physiological functions in regulating HDL metabolism (Glomset et al., 1970; Fotakis et al., 2015), LCAT is being increasingly appreciated in playing a central role in disease, which echoes the fact that Cluster #3 and #9 (cholesterol efflux and reverse cholesterol transport) (Figure 9) has a high concentration of nodes. To summarize the established knowledge, we analyzed the co-citation LCAT-related studies with the top five centralities over the past 35 years (Table 4). Centrality >0.1 means that the citation plays a key role in the co-citation network. From the publication with the highest centrality (van Haperen et al., 2000), they indicated that rapid pre-β-HDL conversion to α-HDL normally carrying most of the circulating plasma HDL cholesterol was driven by LCAT. Besides, other studies shown in Table 4 represented a detailed structural and functional analysis of apoA-I in lipid metabolism (Castro and Fielding, 1988; McManus et al., 2000; Chroni et al., 2003; Nissen et al., 2003). LCAT, which is activated by apoA-I could esterify plasma cholesterol and increase the capacity of HDL to carry and potentially remove cholesterol from tissues, a process referred to as the “RCT” (Cuchel and Rader, 2006). Besides, apoA-I is the primary constituent of HDL which determines the structure and functions of this lipoprotein class and could mediate the cellular synthesis of HDL (Pownall and Ehnholm, 2006). Thus, the balance among LCAT, HDL, and apoA-I is critical to maintaining physiological lipid homeostasis.

### The function and structure of LCAT

Mature LCAT is a glycoprotein with 416 amino acid residues, that is, more hydrophobic compared to plasma lipoproteins. LCAT is intimately involved in the maturation of HDL and LCAT is a key component of the RCT pathway which removes excess cholesterol molecules from the peripheral tissues to the liver for excretion (Ouimet et al., 2019). Glycosylation of LCAT has been shown to be important for its activity and secretion (O et al., 1993) and LCAT is predicted to have an α/β hydrolase fold with Ser181, Asp345 and His377 required for catalytic activity through sequence and mutational analyses (Peelman et al., 1998) and along with two additional subdomains. Subdomain one contains the region of LCAT shown to be required for interfacial activation while subdomain two contains the lid and amino acids that form the back wall of the substrate binding pocket (Piper et al., 2015). LCAT is activated by ApoA-I, which forms a double belt around HDL. There is extensive evidence that the central helices (4–7) of ApoA-I are responsible for LCAT binding and activation, in particular helix 6, which spans residues 143–164 (Sorci-Thomas et al., 2009). Actually, in order to gain new insights into how a phospholipase engages its physiological target and genetic disease influences the interface, a direct visualization of LCAT bound to HDL complexes is needed. It reported that LCAT preferentially interacts with the edge of HDL particles in a manner consistent with making direct interactions with helix 4/6 region of the ApoA-I double belt where LCAT gains access to the acyl tails of lipids at the edge of the protein-delimited lipid bilayer (Manthei et al., 2020). However, because both LCAT and HDLs are highly dynamic entities, it is not the only possible model for the LCAT-HDL complex. Over 90 genetic mutations in LCAT have been described that lead to one of two characterized diseases: fish eye disease and familial LCAT deficiency (Rousset et al., 2009). The mutant residues in the FED form a latching interaction with hydrophobic residues in the lid, making lid displacement difficult (Manthei et al., 2017). Gunawardane et al. (2016) developed an agonistic antibody, 27C3 which binds to and substantially enhances the activity of LCAT from humans and cynomolgus macaques without changing the structure of LCAT. In the LCAT-27C3 crystal complex, the LCAT protein does appear to adopt a more “open” structure with the hydrolase lid pulled away from covering the active site. Therefore, it is very necessary to designed the LCAT-activating drugs target the lid function.

### Alteration of LCAT under physiological conditions

Under physiological conditions, increasing LCAT activity not only enhanced serum HDL level but also induced more functional HDL particles (Tosheska Trajkovska and Topuzovska, 2017). LCAT removes the cholesterol from peripheral macrophage foam cells *via* HDL, and then transports the CE to the liver for excretion through RCT (Badeau et al., 2013). Therefore, maintenance of LCAT activity and concentration are fundamental to sustaining cardiovascular function (Marz et al., 2017; Ouimet et al., 2019). Meanwhile, other physiological states such as exercise could also result in significant alteration of LCAT activity. In endurance athletes, the LCAT activity was increased than that in sedentary men (Gupta et al., 1993). Also, Frey (Frey et al., 1991) reported that LCAT activity was increased after a maximal aerobic stress test in both endurance-trained and sedentary groups. In contrast, it was reported (Williams et al., 1990) that LCAT mass concentrations did not significantly change after a 1-year running program. The reason for the divergent results may be due to the difference in age, physical activity, or metabolic status.

### Alteration of LCAT under pathological conditions

Loss of LCAT function could cause decreased maturation of HDL particles and increased HDL levels of unesterified cholesterol and phosphatidylcholine (Dobiasova and Frohlich, 1998). LCAT deficiency is a rare autosomal recessive disease with an incidence below 1:100, 000 (Lamiquiz-Moneo et al., 2019). The disease is consisting of two main types, FLD and FED. In FLD, both the alpha and beta LCAT activity is lost and result in extremely low serum HDL levels, the premature corneal opacification, hemolytic anemia, proteinuria and renal failure. However, although only the alpha LCAT activity is lost in FED, these individuals could also present with corneal opacities and low HDL levels (Calabresi et al., 2012). Furthermore, the activity and expression of LCAT are dynamic and vary with different pathological conditions.

### Atherosclerotic cardiovascular

Due to the vital role in RCT, variations in LCAT activity seem to be naturally implicated in atherosclerosis prevention or development (Ossoli et al., 2016). Increased LCAT concentration might therefore exert atheroprotective functions in coronary artery disease (CAD) and atherosclerotic cardiovascular disease patients (Hoeg et al., 1996; Ohashi et al., 2005; Rousset et al., 2009).

### Parenchymal liver disease

LCAT is predominantly synthesized by the liver. Studies reported that LCAT participates in the process of nonalcoholic fatty liver disease and liver fibrosis (Nass et al., 2018; Janac et al., 2019). In early studies, LCAT activity tended to be decreased in parenchymal liver disease (Glomset, 1970; Gjone et al., 1971; Cooper et al., 1972; Wengeler et al., 1972). The fibrosis area was negatively correlated with LCAT which suggesting that LCAT could be exploited as a specific biomarker for detecting liver fibrosis (Kawamoto et al., 2006). Previous animal study (Karavia et al., 2013) documented that male LCAT-deficient mice are characterized by increased diet-induced hepatic triglyceride deposition and impaired hepatic histology and architecture, while transgenic mouse and rabbit models with overexpression of the human LCAT gene have a modest reduction in fasting triglyceride (Fruchart and Duriez, 1998). However, recent study showed an increased plasma LCAT activity on average 12% higher in subjects with a fatty liver index (FLI) ≥ 60 as well as in insulin-resistant conditions, like obesity, T2DM, and MetS (Kaya et al., 2013). Hence, elucidating the possible mechanism of the diverse patterns of LCAT activity under different liver pathological conditions is of interest. Moreover, defective liver function could lead to osteoporosis, which known as Hepatic osteodystrophy (HOD), clinically. As a hepatokine, it should also be noted that the potential “organ-crosstalk” regulation of LCAT through circulation. Our recent data (Lu et al., 2022) found that the expression of LCAT was decreased in both HOD patients and mice. Low LCAT level was also associated with low bone mineral density (BMD) and osteoporosis.

### Central nervous system disease

Besides abundantly expressed in the liver, LCAT is also expressed in small amounts in astrocytes in the brain and the testes (Norum, 2017). It was reported that LCAT activity is reduced in cerebrospinal fluid of individuals suffering from Alzheimer’s Disease) (Demeester et al., 2000). And LCAT could esterify both 25-hydroxycholesterol and 27-hydroxycholesterol which were highly neurotoxic, thus preventing the neurotoxic accumulation of oxysterol in the brain (La Marca et al., 2016).

### Reproductive system disease

Similarly, cholesterol is essential for cell membrane stability and the level of cholesterol in seminal plasma was positively associated with semen parameters. LCAT in the epididymis may imply that cholesterol influences the final maturation of spermatozoa (de Neergaard et al., 2018). Moreover, LCAT catalyzes lipid trafficking and reduces oxidative stress that is necessary for the maintenance and repair of oocyte and neuronal membranes and had a significant positive relationship with the fertilization rate (Wu et al., 2016).

### Malignant tumor and other disease

Likewise, malignant tumors often exhibit an altered metabolism of certain lipid metabolizing enzymes compared to normal tissues (Maan et al., 2018). Plasma LCAT showed a decreased activity in colorectal cancer (Mihajlovic et al., 2019) and breast cancer patients (Ozmen and Askin, 2013). And the expression is downregulated in hepatocellular carcinoma (Hu et al., 2020), suggesting that LCAT might involve in the processes of malignancy and prognostic. Also, Russell (Russell et al., 2017) reported that combined biomarker panels (IGFBP2, LCAT, and CA125) provide increased diagnostic lead times for Type I and Type II OC. Moreover, LCAT activity was also decreased in patients with Rheumatoid arthritis and Systemic lupus erythematosus (Charles-Schoeman et al., 2009; Ganjali et al., 2020).

Taken together, under different physiological and pathological conditions, LCAT activity and concentration can be influenced by various transcription factors and then involved in the regulation of lipid homeostasis in the body.

### Emerging trends

Over time, we can see a dynamic development of the LCAT-related studies transition from the structure and function of LCAT (light-colored area) to the relationship with clinical diseases (dark-colored area) as shown in Figure 7. As described previously, abnormal lipid metabolism is associated with diverse disease states. LCAT drives the maturation of nascent HDL into spherical HDL, an essential step in RCT (Zannis et al., 2006). Therefore, deeper insight into the regulation of lipid metabolism mediated by LCAT is needed. Likewise, burst keywords are considered to be indicators of frontier topics or emerging trends and can show the research hotspots during different periods (Li et al., 2021). In this study, the keywords with the strongest citation bursts included “transgenic mice”, “activation”, “human plasma low density”, “hepatic lipase”, “serum lipid”, “identification”, “lipid peroxidation”, “scavenger receptor”, “oxidative stress”, “metabolic syndrome”, “high-density lipoprotein cholesterol “, “risk”, “disease”, “hdl-c level”, “mass spectrometry”, “cardiovascular disease”, “deficiency”, “lecithin: cholestterol acyltransferase”, “cholesterol metabolism”, “familial lcat deficiency”. There were four main research frontiers in terms of burst strength for LCAT-related studies, “transgenic mice”, “oxidative stress”, “risk”, and “cholesterol metabolism”, which attracted the attention of our research group (Figure 8).

The LCAT enzyme is responsible for the production of CE in human plasma (Tosheska Trajkovska and Topuzovska, 2017). Loss of LCAT could lead to serious lipid profile change and thus caused serious concurrent diseases like kidney injury (Hirashio et al., 2014). Likewise, we cannot neglect the possible impact on other organs of low LCAT activity and concentration. As some studies reported, LCAT could serve as a positive risk marker for some diseases. In CAD patients with LCAT mass concentration in the highest quartile had significantly less atheroma burden than those in the lower quartiles which suggested that LCAT was suitable in risk prediction models for atheroma burden (Gebhard et al., 2018). Sethi et al. (2010) also reported that patients with ischemic heart disease have lower LCAT activity, indicating that LCAT is a potentially useful diagnostic marker for cardiovascular disease. Therefore, the “dynamic monitoring” of LCAT activity or concentration together with the identification of disease biomarkers is able to predict the evolution of clinical manifestations and could help the clinician to propose an early diagnosis and to propose an early treatment.

Mechanically, researchers also pay increased attention to the specific mechanism of how LCAT affects disease progression. LCAT synergy with HDL was an important regulator to maintain oxidation and antioxidant balance in the body (Scanu and Edelstein, 2008). In both ApoE^−/−^ and Lcat^−/−^ knock mice, the oxidative stress level showed significantly increased (Ng et al., 2002). In addition, Mertens (Mertens et al., 2003) found that the LCAT activity was lower in double-mutant (Ldlr−/−; ob/ob) mice which showed an impaired antioxidant defense, but after adenovirus-mediated LCAT gene transfer, the plaque volume in the aortic root showed a significant reduction at 6 weeks *via* through upregulation of antioxidant activity. These results indicated that supplement of LCAT could effectively alleviate oxidative stress. Based upon the regulation antioxidant mechanism, additional basic studies are warranted to explore more exactly mechanisms of LCAT regulating the processes of variable diseases. Our recent study (Lu et al., 2022) revealed for the first that LCAT could augment the osteoblast activity and inhibit the osteoclast through regulating cholesterol metabolism. Exogenous supplementation with LCAT also relieve liver fibrosis and increase BMD in the mouse HOD model by promoting the reversal of cholesterol transport from the bone to the liver *via* liver-bone axis. Thus, these discoveries open the possibility that recombinant LCAT may be a treatment for both HOD and liver fibrosis. It’s meaningful to design prophylactic and therapeutic drugs targeting LCAT.

Recently, recombinant human LCAT injections and gene therapy aim to improve plasma lipid metabolism by increasing LCAT activity carried out with promising results which provided better treatment options for patients with LCAT deficiency (Yang et al., 2022). Actually, the recombinant human LCAT (rhLCAT) therapies (http://clinicaltrials.gov NCT04737720) have recently entered clinical phase II trials and have very good prospects. The first use in a human clinical trial was a recombinant human LCAT (designated ACP-501) which was developed by AlphaCore Pharmaceuticals, Inc. In the clinical trial, it was proved that ACP-501 infusion rapidly decreased small- and intermediate-sized HDL, whereas large HDL increased and it was thought well in terms of safety and tolerability in their test results (Shamburek et al., 2016). Besides, another more active rhLCAT (George et al., 2021), MEDI6012, was reported not only reverses HDL dysfunction in ACS patients caused by LCAT concentration/activity but also results in greater HDL function in a 2019 clinical trial (Ossoli et al., 2019). Certainly, intense effort has been focused on developing ways to make a durable and stable plasma LCAT concentrations. With advances in gene therapy, the focus of research and development has shifted to adeno-associated virus (AAV) and lentiviral vectors. Among them, AAV serotype 8 (AAV-8) is the best choice for studying lipoprotein metabolism in mice, with a particularly strong trend towards the liver (Verdera et al., 2020). Guo et al. (2021) have proved that multiple abnormalities such as renal injury, anemia, and atherosclerosis in LCAT-deficient mice were completely rescued after a single injection of AAV-hLCAT. The aforementioned studies demonstrated the potential of LCAT as treatment targets, however, more evidence is still needed to confirm the treatment outcomes of LCAT.

Beyond that, rapid progress has been seen in small molecular activators in recent years. Many peptides which targeted the sequences associated with LCAT activation such as lipid-associated peptide of 20 (LAP-20) (Buchko et al., 1996), amphiphilic Peptide (Manandhar et al., 2017), glutamic acid-alanine-leucine (GALA) (Li et al., 2004), 18 A (Anantharamaiah et al., 1985), and 18A-Pro-18A (Dashti et al., 2004) have been developed to enhance LCAT activity. Small molecule activators of LCAT are good alternative to recombinant enzymes given that cheaper and easier to administer than biologic therapeutics. It was reported that DS-8190a achieved HDL-C elevation in monkey and reduction of atherosclerotic lesion area with enhanced HDL function in rodent through enhancing LCAT activity (Chen et al., 2012). Manthei et al. (2018) reproted a crystal structure of human LCAT in complex with a potent piperidinylpyrazolopyridine activator and an acyl intermediate-like inhibitor which specifically binding to the membrane-binding domain. The compound could stabilize residues in the MBD and facilitate channeling of substrates into the active site without modulating the affinity of LCAT for HDL. Given robust links between structural and functional connectivity in the complex (LCAT, apoA-I and HDL), researchers should also focus on apoA-I, the main activator of LCAT. It was reported that apoA-I is able to activate LCAT *via* displacement of the lid which shield the active site (Manthei et al., 2017). Based on this point, elucidating the mechanistic details behind the activation of LCAT by apoA-I and its mimetic peptides will contribute to several targeted drug delivery applications. In 2015 (Li et al., 2015), reported a 22-residue LCAT-activating peptide (ESP24218) derived from an apoA-I consensus peptide is able to form synthetic HDL particles and activate LCAT to levels similar to apoA-I. However, it remains a challenge to improve the targeting, pharmacokinetic, and dynamic properties of apoA-I mimetic peptides in a manner that makes them more fitting for long-term medication and clinical trials. Reconstituted HDL is a useful vector to study the dynamics of an assembled rHDL:LCAT supramolecular complex. Through generating a rHDL model, Laurenzi (Laurenzi et al., 2021) pointed that the anchoring of LCAT lid to apoA-I helices allows the formation of a hydrophobic hood that expands the LCAT active site and shields it from the solvent, allowing the enzyme to process large hydrophobic substrates. Furthermore, HDL-mimetic nanodiscs are composed of different lipids and apoA-I proteins surrounding lipid bilayers, were composed were and developed clinically for atheroma regression in cardiovascular patients (Patel et al., 2019). Based on a vector of nanodiscs composed of apoA-I mimetic peptides, Giorgi (Giorgi et al., 2022) revealed that peptide 22A forms transient antiparallel dimersin the rim of nanodiscs and the binding strength together with the antiparallel dimerization tendencies of the mimetics can modulate the activity of LCAT. Therefore, future research efforts should also focus on the dynamic interactions and relevant structural patterns among LCAT, apoA-I and HDL which aimed to clarifying the potential clinical therapy of LCAT related small positive allosteric modulators and apoA-I mimetic peptides.

Taken together, further studies should focus on the accurate metabolic process of LCAT dependent or independent of RCT using metabolic marker tracking techniques. It was also well worth to further studying the possibility that LCAT may qualify as a biomarker for risk prediction. Besides, more research efforts should also focus on the dynamic interactions and relevant structural patterns among LCAT, apoA-I and HDL.

## Limitations

It is important to acknowledge that bibliometric studies carry several limitations. Firstly, although WOSCC is the most commonly applied database for scientometric analysis, a few studies not included in WOSCC might be missed. And 1975 was the earliest year we could access to the accessible resources through the database, the literatures published between 1968 and 1975 in our analysis was missed. Besides, this analysis was restricted to international journals in English, therefore a linguistic bias may also exist. Moreover, we used “Lecithin: cholesterol acyltransferase” as a particularly rigorous topic, but we cannot guarantee that each research was completely relevant to the topic. Nevertheless, it is worth mentioning that this study could provide a valid representation within this research field through summarizing the intellectual base and emerging trends.

## Conclusion

Collectively, LCAT-related lipid metabolism studies are in a rapid development stage with active cooperation worldwide. We found that Journal of lipid research published the most LCAT-related studies. Among all the authors who work on LCAT, they tend to collaborate with a relatively stable group of collaborators to generate several major authors clusters which Albers, J. published the most articles (53). And The United States of America had the largest number of publications (1036). LCAT is involved in the processes of several diseases including cardiovascular disease, parenchymal liver disease, central nervous system diseases, cancer, and other lipid metabolism-related diseases through regulating HDL metabolism, thus systematic and dynamical investigation of the difference would elucidate its role in various diseases. Moreover, improved technologies and the increasing application of metabolomics provide the possibility of using LCAT as a biomarker indicating disease diagnosis and progression. This is the first study that demonstrated the trends and future development in LCAT publications, thus offering a brief perspective with regard to future developments in this field.

## Data Availability

The raw data supporting the conclusions of this article will be made available by the authors, without undue reservation.
