# Corrigendum: Influence of lecithin cholesterol acyltransferase alteration during different pathophysiologic conditions: A 45 years bibliometrics analysis

**DOI:** 10.3389/fphar.2022.1133686

**Published:** 2023-01-12

**Authors:** Hongliang Gao, Jing Wu, Zhenyu Sun, Furong Zhang, Tianshu Shi, Ke Lu, Dongfu Qian, Zicheng Yin, Yinjuan Zhao, Jian Qin, Bin Xue

**Affiliations:** ^1^ Core Laboratory, Sir Run Run Hospital, Nanjing Medical University, Nanjing, China; ^2^ School of Clinical Medicine, Wannan Medical College, Wuhu, China; ^3^ Collaborative Innovation Center of Sustainable Forestry in Southern China, College of Forestry, Nanjing Forestry University, Nanjing, China; ^4^ School of Health Policy and Management, Center for Global Health, Nanjing Medical University, Nanjing, China; ^5^ State Key Laboratory of Pharmaceutical Biotechnology, Department of Sports Medicine and Adult Reconstructive Surgery, Nanjing Drum Tower Hospital, The Affiliated Hospital of Nanjing University Medical School, Nanjing, China; ^6^ Research Center for Computer‐Aided Drug Discovery, Chinese Academy of Sciences, Shenzhen, China; ^7^ Nanjing Foreign Language School, Nanjing, China

**Keywords:** lecithin cholesterol acyltransferase, physiological, emerging trends, risk, bibliometrics

In the published article, there was an error in the **Funding** statement. The funding statement for the National Natural Science Foundation of China was displayed as “32071145”.

The correct statement is “*the National Natural Science Foundation of China, 32071142, 32271187*.’’

The authors apologize for this error and state that this does not change the scientific conclusions of the article in any way. The original article has been updated.

