# Supplementary material for: Influence of lecithin cholesterol acyltransferase alteration during different pathophysiologic conditions: A 45 years bibliometrics analysis
Source: Front Pharmacol. 2022 Dec 14;13:1062249. doi: 10.3389/fphar.2022.1062249 (PMC9795195; doi:10.3389/fphar.2022.1062249)
Supplement: Supplementary file 1 [file DataSheet1.PDF]

Supplement table 1. LCAT-related studies published between 1968 and 1975

Rank	Year	Title	PMID	Cited by	Journal	IF
1	1968	The plasma lecithins:cholesterol acyltransferase reaction	4868699	1164	J Lipid Res	6.676
2	1972	A protein cofactor of lecithin:cholesterol acyltransferase	4335615	305	Biochem Biophys Res Commun	2.705
3	1975	Effect of the human plasma apolipoproteins and phosphatidylcholine acyl donor on the activity of lecithin: cholesterol acyltransferase	167813	156	Biochemistry	2.952
4	1973	The metabolic role of lecithin: cholesterol acyltransferase: perspectives form pathology.	4371246	122	Adv Lipid Res	NA
5	1971	Plasma lipoproteins in familial lecithin: cholesterol acyltransferase deficiency: structure of low and high density lipoproteins as revealed by elctron microscopy	5552411	108	J Clin Invest	12.282
6	1970	Physiological role of lecithin-cholesterol acyltransferase.	4927041	70	Am J Clin Nutr	8.472
7	1969	Anemia with spur cells: a red cell defect acquired in serum and modified in the circulation	5822588	68	J Clin Invest.	12.282
8	1972	Lecithin: cholesterol acyltransferase: effects of substrate composition upon enzyme activity	4340992	54	Biochim Biophys Acta	NA
9	1968	In vitro incorporation of cholesterol-14C into very low density lipoprotein cholesteryl esters	5726322	53	J Lipid Res	4.743
10	1970	Activation of lipoprotein lipase by lipoprotein fractions of human serum	5534018	52	J Lipid Res	4.743
11	1973	The metabolic role of lecithin: cholesterol acyltransferase: perspectives form pathology	4354500	51	Adv Lipid Res	NA
12	1973	Lecithin: cholesterol acyltransferase of human plasma. Role of chylomicrons, very low, and high density lipoproteins in the reaction	4356623	44	J Biol Chem	4.106
13	1970	Plasma lecithin-cholesterol acyltransferase and erythrocyte lipids in liver disease	5444972	42	Acta Med Scand	NA
14	1971	Plasma lecithin: cholesterol acyltransferase activity in liver disease	5558781	39	Eur J Clin Invest	2.784
15	1975	The hypercholesterolaemia of obstructive jaundice	166902	35	Gut.	17.943
16	1972	Identification of the abnormal cholestatic lipoprotein (LP-X) in familial lecithin:Cholesterol acyltransferase deficiency	11946663	32	FEBS Lett	2.675
17	1974	Phase transitions in bilamellar vesicles. Measurements by pyrene excimer fluorescence and effect on transacylation by lecithin: cholesterol acyltransferase	4407847	31	Biochemistry	2.952
18	1973	Positive correlation of serum lecithin: cholesterol acyltransferase activity with relative body weight	4696509	29	Eur J Clin Invest	2.784
19	1972	Exchange of phospholipids between low and high density lipoproteins of squirrel monkeys	4335797	28	J Lipid Res	4.743
20	1972	Observations on the lecithin: cholesterol acyltransferase system in bovine plasma	5054303	27	Biochim Biophys Acta	NA
21	1969	Familial plasma lecithin: cholesterol acyltransferase deficiency	5780456	26	Br Med J	NA
22	1975	Lipoproteins and lipid transport	173151	26	Adv Exp Med Biol	2.126

23	1975	Evaluation of an in vitro assay of lecithin:cholesterol acyl transfer rate in plasma	1209161	26	Scand J Clin Lab Invest	1.38
24	1972	Serum cholesterol esterification in liver disease. Combined determinations of lecithin: cholesterol acyltransferase and lipoprotein-X	5082071	25	Eur J Clin Invest	2.784
25	1973	Lecithin: cholesterol acyl-transferase and lipoprotein-X in liver disease	4772663	25	Clin Chim Acta	2.735
26	1975	Lecithin:cholesterol acyl transfer in plasma of normal persons in relation to lipid and lipoprotein concentration	174186	25	Scand J Clin Lab Invest	1.38
27	1974	Possible association between an abnormal low density lipoprotein and nephropathy in lecithin: cholesterol acyltransferase deficiency	4367873	24	Clin Chim Acta	2.735
28	1973	A method for the determination of the initial rate of reaction of lecithin: cholesterol acyltransferase in human plasma	4353708	22	Biochim Biophys Acta.	NA
29	1972	Sea-blue histiocytes in familial lecithin: cholesterol acyltransferase deficiency	4114809	21	Scand J Haematol	NA
30	1971	Red cell lipids in liver disease: relationship to serum lipids and to lecithin-cholesterol acyltransferase	5559858	21	J Lab Clin Med	NA
31	1970	Familial lecithin: cholesterol acyltransferase deficiency. Study of two new patients and their close relatives	5479662	20	Acta Med Scand	NA
32	1971	The relationship between serum lipoproteins and red cell membranes in abetalipoproteinemia: deficiency of lecithin:cholesterol acyltransferase	5092855	20	J Lab Clin Med	NA
33	1971	Origin and disappearance of plasma lecithin: cholesterol acyltransferase	5545684	19	Am J Physiol	NA
34	1974	Lecithin:cholesterol acyltransferase and plasma proteins in liver diseases	4366882	19	Clin Chim Acta	2.735
35	1972	The effects of unsaturated and saturated dietary fats on plasma cholesterol, phospholipids and lecithin: cholesterol acyltransferase activity	5064529	17	Acta Med Scand	NA
36	1975	Lipoprotein and lecithin: cholesterol acyltransferase changes in galactosamine-induced rat liver injury	812181	16	Science	41.037
37	1970	Production of lecithin: cholesterol acyltransferase by the isolated perfused rat liver	5499211	15	Biochim Biophys Acta.	NA
38	1968	A method for studying the interaction between lecithin: cholesterol acyltransferase and high density lipoproteins	5682287	15	Biochem Biophys Res Commun	2.705
39	1975	Cholesterol esterification and lipids in plasma and liver from newborn and young guinea pigs raised on milk and non-milk diet	1187072	15	Nutr Metab	NA
40	1971	Use of sonicated dispersions of mixtures of cholesterol with lecithin as substrates for lecithin:cholesterol acyltransferase	5546578	13	Biochim Biophys Acta	NA
41	1974	Familial lecithin:cholesterol acyltransferase deficiency. Studies on lipid composition and morphology of tissues	4367608	13	Scand J Clin Lab Invest Suppl	NA
42	1971	Plasma lecithin: cholesterol acyltransferase activity in acute hepatitis	5572894	13	Scand J Gastroenterol	2.152
43	1972	Origin of cholesteryl esters in the blood of cholesterolfed rabbits: relative contributions of serum lecithin-cholesterol acyltransferase and hepatic ester synthesis	5012982	13	Biochim Biophys Acta	NA

44	1974	Familial lecithin:cholesterol acyltransferase deficiency--a clinical survey	4367606	12	Scand J Clin Lab Invest Suppl	NA
45	1973	Does lipoprotein-X (LP-X) act as a substrate for the lecithin: cholesterol acyltransferase (LCAT)?	4730213	12	Clin Chim Acta	2.735
46	1968	Effect of hypercholesteremia on the activity of serum lecithin-cholesterol acyltransferase	17805892	12	Lipids.	2.144
47	1973	Identification of lipoprotein families in familial lecithin: cholesterol acyltransferase deficiency	4359868	12	Biochim Biophys Acta	NA
48	1974	Lecithin-cholesterol acyltransferase activity in diabetes mellitus and the effect of insulin on these cases	4214409	11	Clin Chim Acta	2.735
49	1975	Substrate specificity of lecithin:cholesterol acyltransferase. Esterification of desmosterol, b-sitosterol, and cholecalciferol in human plasma	174187	11	Scand J Clin Lab Invest	1.38
50	1973	Lecithin-cholesterol acyltransferase and serum cholesterol esterification in obstructive jaundice	4683419	11	J Lab Clin Med	NA
51	1970	Plasma lecithin-cholesterol acyltransferase in obstructive jaundice	5429895	11	Scand J Gastroenterol	2.152
52	1973	Observations on changes in lipid composition and lecithin-cholesterol-acyltransferase reaction of bovine plasma induced by heat exposure	4713742	10	Lipids	2.144
53	1973	Effect of lysolecithin and albumin on lecithin-cholesterol acyltransferase activity in human plasma	4360812	9	J Biochem	2.23
54	1975	Lipoproteins in lecithin-cholesterol-acyltransferase(LCAT)-deficiency. II. Further studies on the abnormal high-density-lipoproteins	168146	9	Humangenetik	NA
55	1973	Familial plasma lecithin: cholesterol acyltransferase (LCAT) deficiency. Ultrastructural aspects of a new syndrome with particular reference to lesions in the kidneys and the spleen	4771974	9	Acta Pathol Microbiol Scand A	NA
56	1974	The ultrastructure of plasma lipoproteins in lecithin:cholesterol acyltransferase deficiency	4136021	9	Scand J Clin Lab Invest Suppl.	NA
57	1973	Role of high density lipoproteins in the lecithin-cholesterol acyltransferase activity with sonicated lecithin-cholesterol dispersions as substrate	4347393	9	Biochim Biophys Acta	NA
58	1973	Inhibition of lecithin: cholesterol acyltransferase following intravenous administration of heparin in man	4776440	9	Biochim Biophys Acta	NA
59	1968	The effect of plasma transfusion on the plasma cholesterol esters in patients with familial plasma lecithin: cholesterol acyltransferase deficiency	5741800	8	Scand J Clin Lab Invest	1.38
60	1975	Synthesis of cholesterol esters in the plasma and liver of sheep	173965	8	Lipids	2.144
61	1968	The influence of plasma from patients with familial plasma lecithin: cholesterol acyltransferase deficiency on the lipid pattern of erythrocytes	5710162	8	Scand J Clin Lab Invest	1.38
62	1969	Presence of (alpha)-1-lipoprotein in patients with familial plasma lecithin: cholesterol acyltransferase deficiency	4986447	8	Scand J Clin Lab Invest	1.38
63	1974	Serum cholesterol esterification in human liver disease: role of lecithin-cholesterol acyltransferase and cholesterol ester hydrolase	4856585	7	Gastroenterology	19.233
64	1975	Changes in plasma lipoprotein constituents during constant infusions of heparin	1201153	7	Atherosclerosis	NA

65	1975	Lecithin:cholesterol acyltransferase activity in hypercholesterolemic subjects and in hypercholesterolemic subjects treated with clofibrate	1207415	6	Lipids	2.144
66	1969	Various factors affecting cholesterol esterification in plasma lipoproteins by lecithin-cholesterol acyltransferase	5800047	6	Biochim Biophys Acta	NA
67	1975	Effect of lipid emulsions on the plasma lecithin: cholesterol acyltransfer in guinea pigs	1241794	6	6 Nutr Metab.	NA
68	1975	The relationship of red cell membrane lipid content to red cell morphology and survival in patients with liver disease	1057918	6	Aust N Z J Med	NA
69	1974	[Lecithin degradation and esterification of cholesterol in human serum. Further results on the role of lecithin-cholesterol acyltransferase in lipid metabolism under normal and pathological conditions (author's transl)]	4599334	5	Klin Wochenschr	NA
70	1971	Lecithin: cholesterol acyltransferase activity in the plasma of rats fed sucrose or starch	5127158	5	Nutr Metab	NA
71	1974	Effect of organophosphate pesticides on lecithin-cholesterol acyltransferase in human plasma	4847537	5	Biochem Pharmacol	4.825
72	1972	Studies on the protein moiety of serum high density lipoprotein from patients with familial lecithin: cholesterol acyltransferase deficiency	4341063	5	Clin Genet	4.104
73	1973	Ultrastructural aspects of familial lecithin-cholesterol acyltransferase deficiency	4711720	4	Nutr Metab	NA
74	1974	Plasma lecithin: cholesterol acyltransferase activity in hypo- and hyperthyroidism	4828274	4	Horm Metab Res	2.423
75	1973	A stable plasma lipoprotein and lecithin cholesterol acyltransferase control material	4351091	4	Clin Chim Acta	2.735
76	1974	Plasma lipoproteins in patients with familial plasma lecithin: cholesterol acyltransferase (LCAT) deficiency--studies on the apolipoprotein composition of isolated fractions with identification of LP-X	4371225	4	Horm Metab Res	2.423
77	1971	Effects of pure oxygen atmosphere in vivo on plasma lecithin-cholesterol acyltransferase reaction	4400984	4	Lipids	2.144
78	1974	Lipid and lipoprotein abnormalities in liver disease: the possible role of lecithin: cholesterol acyltransferase deficiency	4367610	3	Scand J Clin Lab Invest Suppl	NA
79	1975	Lecithin:cholesterol acyltransferase initial fractional rates of esterification in human and rat serum during development	1192610	3	Clin Chim Acta	2.735
80	1972	Inhibition of the plasma lecithin-cholesterol acyltransferase reaction by hydrogen peroxide and peroxidized lecithin	5012338	3	Lipids	2.14
81	1971	Familial plasma lecithin: cholesterol acyltransferase deficiency. A study of the platelets	5581187	3	Scand J Clin Lab Invest	1.38
82	1974	Studies on enzymatic and molecular properties of lecithin:cholesterol acyltransferase	4367599	3	Scand J Clin Lab Invest Suppl	NA
83	1974	Lecithin: cholesterol acyltransferase recent research on biochemistry and physiology of the enzyme	4604673	2	Scand J Clin Lab Invest	2.209

84	1973	Lecithin-cholesterol-acyltransferase (LCAT) activity in hyper- and hypothyroidism	4542075	2	Acta Endocrinol Suppl (Copenh)	NA
85	1974	Studies on the cofactor requirements for lecithin:cholesterol acyltransferase	4845395	2	Scand J Clin Lab Invest Suppl	NA
86	1974	Ocular manifestations in familial lecithin: cholesterol acyltransferase deficiency	4845396	2	Scand J Clin Lab Invest Suppl.	NA
87	1974	Plasma lipoprotein metabolism in familial lecithin:cholesterol acyltransferase deficiency	4367614	2	Scand J Clin Lab Invest Suppl	NA
88	1974	Apolipoproteins and lipoprotein families in familial lecithin: cholesterol acyltransferase deficiency	4367607	2	Scand J Clin Lab Invest Suppl	NA
89	1975	Depression of lecithin-cholesterol acyltransferase esterification in vitamin E-deficient monkeys	808956	1	Am J Clin Nutr	6.568
90	1974	Lecithin:cholesterol acyltransferase in human liver disease	4845397	1	Scand J Clin Lab Invest Suppl	NA
91	1972	Plasma lecithin/cholesterol-acyltransferase activity in multiple sclerosis	4113212	1	Lancet	59.102
92	1974	In vitro determination of lecithin:cholesterol acyltransferase in plasma	4367600	1	Scand J Clin Lab Invest Suppl	NA
93	1973	The role of lecithin: cholesterol acyltransferase in the metabolism of plasma lipoproteins	4356109	1	Ann Biol Clin (Paris)	0.503
94	1972	[Lecithin-cholesterol-acyltransferase system in human plasma in liver parenchyma diseases]	4665553	1	Verh Dtsch Ges Inn Med	NA
95	1969	The plasma lecithin: cholesterol acyltransferase reaction in normal, hypophysectomized and thyroidectomized rats and in hamsters	5777630	1	Life Sci	3.448
96	1975	Effect of ozone exposure on lecithin : cholesterol acyltransferase activity in rat serum	1240854	1	Hokkaido Igaku Zasshi	NA
97	1973	Plasma lecithin-cholesterol acyltransferase deficiency in a child with terminal pulmonary hyaline membrane disease	4800877	1	Arch Dis Child	3.158
98	1974	Effect of lysophosphatidyl choline on interaction between phosphatidyl choline and activator protein (apolipoprotein A-I) of lecithin:cholesterol acyltransferase	4136022	1	Scand J Clin Lab Invest Suppl	NA
99	1972	ABO blood groups, serum cholesteryl esters and plasma lecithin: cholesterol acyltransferase activity	5054809	1	Clin Genet	4.104
100	1973	[Dependence of inhibition of an accelerated blood sedimentation rate from lecithin-cholesterol-acyltransferase system activity in human plasma]	4790155	1	Verh Dtsch Ges Inn Med	NA
101	1975	Effect of simian virus 40 subcutaneous tumors on circulating lipids and lipoproteins in the Syrian hamster	163332	1	J Natl Cancer Inst	10211
102	1974	[Familial lecithin-cholesterol-acyltransferase-deficiency]	4822294	0	Ugeskr Laeger	NA
103	1970	Plasma lipoproteins in familial lecithin: cholesterol acyltransferase deficiency: lipid composition and reactivity in vitro	5456796	0	J Clin Invest	12.282
104	1975	Genetics of LCAT (lecithin: cholesterol acyltransferase) deficiency	806250	0	Ann Hum Genet	1.319
105	1971	Action of lecithin-cholesterol acyltransferase on sonicated dispersions of lecithin and cholesterol and on lecithin-cholesterol-protein complexes	5000278	0	Biochim Biophys Acta	NA

106	1975	Quantitative studies of lipoprotein-X in familial lecithin: cholesterol acyltransferase deficiency and during cholesterol esterification	1091378	0	Clin Chim Acta	2.735
107	1973	Plasma lipoproteins in familial lecithin: cholesterol acyltransferase deficiency. Further studies of very low and low density lipoprotein abnormalities	4349484	0	J Clin Invest	12.282
108	1975	Lipoprotein metabolism	174409	0	Adv Lipid Res	NA
109	1971	Plasma lipoproteins in familial lecithin: cholesterol acyltransferase deficiency: physical and chemical studies of low and high density lipoproteins	5552410	0	J Clin Invest	12.282
110	1975	Plasma lipoproteins in familial lecithin: cholesterol acyltransferase deficiency: effects of dietary manipulation	169566	0	Scand J Clin Lab Invest Suppl	NA
111	1971	Interaction of lecithin:cholesterol acyltransferase with sonicated dispersions of lecithin	5546579	0	Biochim Biophys Acta	NA
112	1975	Plasma lipoproteins in familial lecithin: cholesterol acyltransferase deficiency: effects of incubation with lecithin: cholesterol acyltransferase in vitro	169567	0	Scand J Clin Lab Invest Suppl	NA
113	1974	Familial lecithin:cholesterol acyltransferase deficiency. Ultrastructural studies on lipid deposition and tissue reactions	4845398	0	Scand J Clin Lab Invest Suppl	NA
114	1972	[Research on serum lecithin-cholesterol-acyltransferase]	4576218	0	Bull Acad Natl Med	NA
115	1974	Lecithin: cholesterol acyltransferase of human plasma. Role of chylomicrons, very low, and high density lipoproteins in the reaction	4367601	0	Scand J Clin Lab Invest Suppl	NA
116	1974	[Distribution of cholesterol and activity of lecithin-cholesterol--acyltransferase (LCAT) in quail, as a function of sex]	4368108	0	C R Acad Hebd Seances Acad Sci D	NA
117	1975	[Influence of radiation on plasma lecithin-cholesterol acyltransferase in rats]	126783	0	C R Seances Soc Biol Fil	NA
118	1974	Current concepts of cholesterol metabolism and their relationship to lecithin:cholesterol acyltransferase	4367613	0	Scand J Clin Lab Invest Suppl.	NA
119	1975	[Lecithin cholesterol acyltransferase (LCAT) of various vascular sections in man]	1226987	0	Verh Dtsch Ges Inn Med	NA
120	1972	[Determination of serum-lecithin-cholesterol-acyltransferase enzyme (LCAT)]	5035528	0	Z Med Labortech	NA
121	1970	[Lecithin: cholesterol acyltransferase reaction in chicken serum. 2. Alteration of serum lipoproteins during incubation]	5464866	0	Seikagaku	NA
122	1970	[Lecithin: cholesterol acyltransferase reaction in chicken serum. I. Effect of inorganic salts]	5464865	0	Seikagaku	NA
123	1974	Familial lecithin:cholesterol acyltransferase deficiency. Report of a third Norwegian family with two afflicted members	4367609	0	Scand J Clin Lab Invest Suppl	NA
124	1973	[Activity of lecithin-cholesterol-acyltransferase in patients with arteriosclerosis]	4795177	0	Ter Arkh	0.359
125	1973	The lipemia in familial plasma lecithin: cholesterol acyltransferase deficiency	5507244	0	Acta Med Scand	NA

126	1972	[Hyperlipemia and plasma lecithin-cholesterol acyltransferase activity]	4674489	0	Nihon Ronen Igakkai Zasshi	NA
127	1971	[Serum lipoprotein and lecithin cholesterol acyltransferase]	5167906	0	Rinsho Byori.	NA
128	1974	Structural studies on serum lipoproteins in homozygotes and heterozygotes for the lecithin:cholesterol acyltransferase deficiency gene	4367611	0	Scand J Clin Lab Invest Suppl	NA
129	1974	Phospholipid substrate specificity of purified human plasma lecithin:cholesterol acyltransferase	4367597	0	Scand J Clin Lab Invest Suppl	NA
130	1971	Amino acid composition of serum high density lipoprotein in patients with familial lecithin:cholesterol acyltransferase deficiency	5116595	0	Clin Genet.	4.104
131	1975	Membrane structure and its relation to haemolysis	810289	0	Clin Haematol	NA
132	1972	Plasma lipoproteins in patients with familial plasma lecithin: cholesterol acyltransferase deficiency: apolipoprotein composition of isolated fractions	4665520	0	Verh Dtsch Ges Inn Med	NA
133	1975	[Effect of essential phospholipids (EPL) on plasma lecithin-cholesterol acyltransferase (LCAT) activity in vivo and in vitro in the rabbit]	1226995	0	Verh Dtsch Ges Inn Med	NA
134	1973	[Investigation of lecithin-cholesterol-acyltransferase (LACT) activity in the serum of three patients with Tangier disease (author's transl)]	4359072	0	Klin Wochenschr.	NA
135	1975	Human growth hormone as an activator for lecithin--cholesterol acyl-transferase	1175251	0	Chem Phys Lipids	2.536
136	1974	[Mechanism of blood sedimentation. XVII. Relationship and diagnostic importance of lecithin-cholesterol-acyltransferase (LCAT) activity in human serum and heat-induced inhibition of erythrocyte sedimentation (author's transl)]	4855484	0	Klin Wochenschr	NA
137	1969	The selective and conjoint loss of red cell lipids	5780200	0	J Clin Invest	12.282
138	1974	[Symposium on hyperlipidemia--epidemiological, clinical and experimental studies. Plasma lipoprotein metabolism and lecithin: cholesterol acyltransferase (author's transl)]	4474332	0	Nihon Naika Gakkai Zasshi	NA
139	1972	An analysis of lipoproteins, bile acids, and red cell membranes associated with target cells and spur cells in patients with liver disease	4640953	0	J Clin Invest.	12.282
140	1975	Stimulation of cholesterol ester exchange by lipoprotein-free rabbit plasma	173390	0	Biochim Biophys Acta	NA
141	1975	Metabolism of cholesteryl esters of rat very low density lipoproteins	166092	0	J Clin Invest	12.282
142	1972	[Lecithin: cholesterol acyl-transferase activity in the plasma of patients with multiple sclerosis]	5042254	0	Acta Neurol (Napoli)	NA
143	1975	Lecithin cholesterol acyl transferase deficiency. Light and electron microscopic finding from two corneas	811335	0	Can J Ophthalmol	1.305
144	1972	Characterization of guinea pig plasma lipoproteins: the appearance of new lipoproteins in response to dietary cholesterol	4342426	0	J Lipid Res	4.743
145	1970	Metabolism of lysolecithin in vivo: effects of hyperlipemia and atherosclerosis in squirrel monkeys	4994121	0	J Lipid Res	4.743

146	1974	[Hereditary disorders of cholesterol metabolism (literature survey)]	4219824	0	Vopr Okhr Materin Det	NA
147	1975	The relationship between dietary fat composition and plasma cholesterol esterification in man	1204293	0	Clin Sci Mol Med	NA
148	1974	Lipoproteins in a nonrecirculating perfusate of rat liver	4372284	0	J Lipid Res	4.743
149	1974	[Gas-chromatographic method for determination of plasma lecithin-cholesterol acyl transferase (LCAT) activity]	4460077	0	Quad Sclavo Diagn	NA
150	1975	Coil planet centrifugation and its application to the observation of altered membrane properties of erythrocytes in hepatobiliary disorders	1123561	0	J Lab Clin Med	NA
151	1975	Letter: Primary L.C.A.T.-deficiency disease	47584	0	Lancet	59.102
152	1975	Plasma cholesterol ester formation in the neonatal lamb	1148339	0	Biol Neonate	NA
153	1975	Inhibition of cholesterol esterification by polyene antibiotics in blood plasma	1122313	0	Biochim Biophys Acta	NA
154	1975	Plasma lipoprotein abnormalities in a case of primary high-density lipoprotein (HDL) deficiency	171109	0	Clin Genet	4.104
155	1975	Diagnostic importance of lipoprotein-X (LP-X) in the diagnosis of cholestasis	175222	0	Mater Med Pol.	NA
156	1975	[Physiology of lipids. Qualitative and quantitative mechanisms of liporegulation]	1178131	0	Minerva Med	NA
